# Novel *LTBP3* mutations associated with thoracic aortic aneurysms and dissections

**DOI:** 10.1186/s13023-021-02143-2

**Published:** 2021-12-14

**Authors:** Guoyan Zhu, Mingyao Luo, Qianlong Chen, Yinhui Zhang, Kun Zhao, Yujing Zhang, Chang Shu, Hang Yang, Zhou Zhou

**Affiliations:** 1 State Key Laboratory of Cardiovascular Disease, Beijing Key Laboratory for Molecular Diagnostics of Cardiovascular Diseases, Diagnostic Laboratory Service, Fuwai Hospital, National Center for Cardiovascular Diseases, Chinese Academy of Medical Sciences and Peking Union Medical College, Beijing, 100037 China; 2 State Key Laboratory of Cardiovascular Disease, Center of Vascular Surgery, Fuwai Hospital, National Center for Cardiovascular Diseases, Chinese Academy of Medical Sciences and Peking Union Medical College, Beijing, 100037 China

**Keywords:** Thoracic aortic aneurysm and dissection, *LTBP3* gene, Genetic mutation

## Abstract

**Background:**

Thoracic aortic aneurysm and dissection (TAAD) is a hidden-onset but life-threatening disorder with high clinical variability and genetic heterogeneity. In recent years, an increasing number of genes have been identified to be related to TAAD. However, some genes remain uncertain because of limited case reports and/or functional studies. *LTBP3* was such an ambiguous gene that was previously known for dental and skeletal dysplasia and then noted to be associated with TAAD. More research on individuals or families harboring variants in this gene would be helpful to obtain full knowledge of the disease and clarify its association with TAAD.

**Methods:**

A total of 266 TAAD probands with no causative mutations in known genes had been performed wholeexome sequencing (WES) to identify potentially pathogenic variants. In this study, rare *LTBP3* variants were the focus of analysis.

**Results:**

Two compound heterozygous mutations, c.625dup (p.Leu209fs) and c.1965del (p.Arg656fs), in *LTBP3* were identified in a TAAD patient along with short stature and dental problems, which was the first TAAD case with biallelic *LTBP3* null mutations in an Asian population. Additionally, several rare heterozygous *LTBP3* variants were also detected in other sporadic TAAD patients.

**Conclusion:**

The identification of *LTBP3* mutations in TAAD patients in our study provided more clinical evidence to support its association with TAAD, which broadens the gene spectrum of *LTBP3*. *LTBP3* should be considered to be incorporated into the routine genetic analysis of heritable aortopathy, which might help to fully understand its phenotypic spectrum and improve the diagnostic rate of TAAD.

## Background

Thoracic aortic aneurysms are often asymptomatic, as they progressively enlarge and are finally aware of being diagnosed clinically when dissections or ruptures occur, which are life-threatening and can cause sudden death in up to 50% of patients [1, 2]. Therefore, early diagnosis and timely treatment of thoracic aortic aneurysm and dissection (TAAD) are very important for reducing mortality.

Many factors contribute to TAAD development, while genetic defects play a major role. Marfan syndrome (MFS) is the most well-known heritable aortic disease, and other syndromic connective diseases involving the aorta, such as Loeys–Dietz syndrome (LDS) and vascular Ehlers–Danlos syndrome (EDS), are less commonly seen. An increasing number of causative mutations have also been identified in nonsyndromic TAAD patients, but they can only explain a small proportion (approximately 20%) of TAAD families [3], which suggests that there are many unrecognized genes to be explored.

To date, 11 genes (*ACTA2, COL3A1, FBN1, LOX, MYH11, MYLK, PRKG1, SMAD3, TGFB2, TGFBR1, TGFBR2*) have been confirmed to be responsible for heritable TAAD [1]. These genes encode proteins involved in smooth muscle cell contraction, the extracellular matrix, and transforming growth factor-beta (TGF-β) signaling [4]. Several genes, such as *BGN, FOXE3, HCN4, MAT2A, MFAP5, SMAD2*, and *TGFB3*, remain uncertain [1] because they were recently discovered, and there have been few reported cases and insufficient evidence. However, as research continues, their association with TAAD may become clear.

*LTBP3*, which encodes an extracellular matrix protein, is highly expressed in human ovaries, prostate, fat, heart, and skeletal muscle [5, 6]. Mutations in this gene were previously known to be associated with skeletal dysplasia, such as dental anomalies and short stature (DASS; OMIM#601216), geleophysic dysplasia 3 (GPHYSD3; OMIM#617809), and acromicric dysplasia (ACMICD; OMIM #102370) [7–9]. Until in 2018, *LTBP3* was firstly reported to predispose individuals to TAAD [10]. Although the underlying mechanisms are not fully elucidated, the involvement of *LTBP3* in TAAD is not unexpected considering its close association with *FBN1*, the MFS gene [11, 12].

*LTBP3* belongs to the latent TGF-ß binding protein (LTBP) family and contains several modules including TGF-β-binding (TB) domain, epidermal growth factor (EGF), 4 Cys, hybrid, and calcium-binding EGF domains [13], which closely resembles that of fibrillin 1. Studies have shown that LTBP3 is assisted by fibrillin-1 to incorporate into the extracellular matrix, which can be hindered in the absence of fibrillin-1 microfibrils in vivo or in vitro [12]. Meanwhile, it has an essential role in regulating the TGF-β signaling pathway [5, 6]. Therefore, LTBP3 might be involved in TAAD. Nevertheless, only a few cases with *LTBP3* mutations have been reported and we may not understand the full clinical spectrum of the disease. Aortic deformity might be an important characteristic that occurs more frequently but has been neglected in the past.

In this study, we reported two compound heterozygous *LTBP3* mutations in a TAAD patient along with DASS and several rare heterozygous *LTBP3* variants in sporadic TAAD patients. Our results provide more clinical evidence supporting that *LTBP3* mutations might be responsible for heritable TAAD.

## Materials and methods

### Patients

Patients with TAAD from the Center of Vascular Surgery in Fuwai Hospital were referred to our Diagnostic Laboratory Service for genetic testing. The targeted sequencing contained 15 genes (*FBN1, TGFB2, SMAD3, TGFBR1, TGFBR2, ACTA2, MYH11, SMAD4, MYLK, NOTCH1, PRKG1, SKI, COL3A1, SLC2A10, FBN2*) associated with aortic disease and was performed as previously reported [14]. Among them, 266 patients who did not have an identified causative mutation in the panel testing were recruited in our study. Wholeexome sequencing was then performed to identify potentially pathogenic mutations.

## Whole exome sequencing (WES)

Genomic DNA was extracted from EDTA-anticoagulated whole blood of patients and their relatives using a QIAamp DNA Blood Mini Kit (Qiagen, Hilden, Germany) according to the manufacturer’s instructions [15, 16]. The DNA samples and sequencing data were processed as previously described [17]. WES was performed on an Illumina HiSeq2500 platform (Illumina Inc., San Diego, CA, USA) using the TruSeq Rapid PE Cluster kit V2 or TruSeq Rapid SBS kit V2 - HS (Illumina Inc., San Diego, CA, USA).

## Bioinformatics analysis

The process of WES bioinformatics analysis can be roughly divided into three modules: data preprocessing, variant detection, and annotation. FastP software was used to filter the low-quality sequence reads [18], and sequence reads were aligned to the reference genome hg19 using BWA (Burrows–Wheeler Aligner) MEM software [15, 19, 20]. Picard (MarkDuplicates) software (https://github.com/broadinstitute/picard) was used to label repetitive reads, and GATK3.7 software was used to correct systematic errors in the sequencing process. The GATK Haplotypecaller (https://gatk.broadinstitute.org) was used in variant calling, and Annovar software (https://annovar.openbioinformatics.org/en/latest/) was used for annotation analysis. High quality variants (mean sequencing depth >=20×, alternation frequency (AltFeq) between 0.3 and 0.6 or > 0.95, strand bias (STB) between 0.5 and 0.7) detected by WES were filtered for further analysis. Variants based on the < 1% minor allele frequency (MAF) in any gnomAD (version 2.1) population (gnomAD_popmax) and location in the coding regions and variable splicing sites (canonical ±1 or 2 splice sites or splice sites of either dbscSNV_ADA or dbscSNV_RF > 0.6 were retained. Then *LTBP3* rare variants with a MAF < 0.01 and < 0.00005 in gnomAD were focused separately for autosomal recessive and dominant conditions. The variant interpretation was then performed according to ACMG guidelines [21, 22]. Variants detected in our study were written according to the HGVS nomenclature rules.

## Sanger sequencing

All the mutations detected by WES were verified by Sanger sequencing. According to the reference genomic sequences of the Human Genome from GenBank in NCBI [22], Primer3 Input(version 0.4.0) was used to design primer pairs. Sanger sequencing was performed with the following primers:

**Table:** 

Gene	Forward primer	Reverse primer
*LTBP3_*Exon1	CGGCCCTCTACTCCCTTC	GTCCGCTTGCAGATCACC
*LTBP3_*Exon2	GAGGAGGGGAAAGAGACAGG	GGCGTTCGAGCTCTCAAT
*LTBP3_*Exon8	CACCGGTGAGTCAGGGTTAC	TTGGGGGTTAGACTGTGAGG
*LTBP3_*Exon10	ACTTCATGGCCCCATCTTCT	CCCAGTGATTTAGCCCTTGA
*LTBP3*_Exon13	CTTGGCCTACCCGTTCTTCT	AGTGACCGGGAAAGTTGATG

## Results

Homozygous or compound heterozygous *LTBP3* mutations were first reviewed in our 266 WES data, with a MAF< 0.01 in gnomAD. Two heterozygous mutations, c.625dup (p.Leu209fs) and c.1965del (p.Arg656fs), were detected in patient AD2002. These two variants were not recorded in the population database, and both of them were predicted to undergo nonsense-mediated decay. They were both confirmed by Sanger sequencing. After detecting his parents’ genotype, we found that one of them (c.1965del, p.Arg656fs) was inherited from his mother, while the other (c.625dup, p.Leu209fs) was de novo. To determine whether they were in *trans* or *cis*, the genotypes of their offspring were also detected. His twin sons carried only the variant c.625dup, which highly suggested that these two variants were on opposite chromosomes. Therefore, these compound heterozygous variants could be interpreted as pathogenic according to the ACMG guidelines (Table 1) and were causative for his disease.

**Table 1:** Compound heterozygous mutations in *LTBP3* identified in patient AD2002

Mutations	Inheritance	MAF	Pathogenicity	ACMG evidence	Genotype of relatives
*LTBP3*: NM_001130144: c.1965del (p.Arg656Alafs*6)	Autosomal recessive	0	Pathogenic	PVS1, PM2, PM3	Father: −/− Mother: +/− Sister: +/− Elder son: −/− Youngest son: −/−
*LTBP3*: NM_001130144: c.625dup (p.Leu209Profs*38)	Autosomal recessive	0	Pathogenic	PVS1, PS2, PM2, PM3	Father:−/− Mother:−/− Sister: −/− Elder son: +/− Youngest son: +/−

MAF, minor allele frequence; −/−, wild type; +/−, heterozygous

**Fig. 1:**
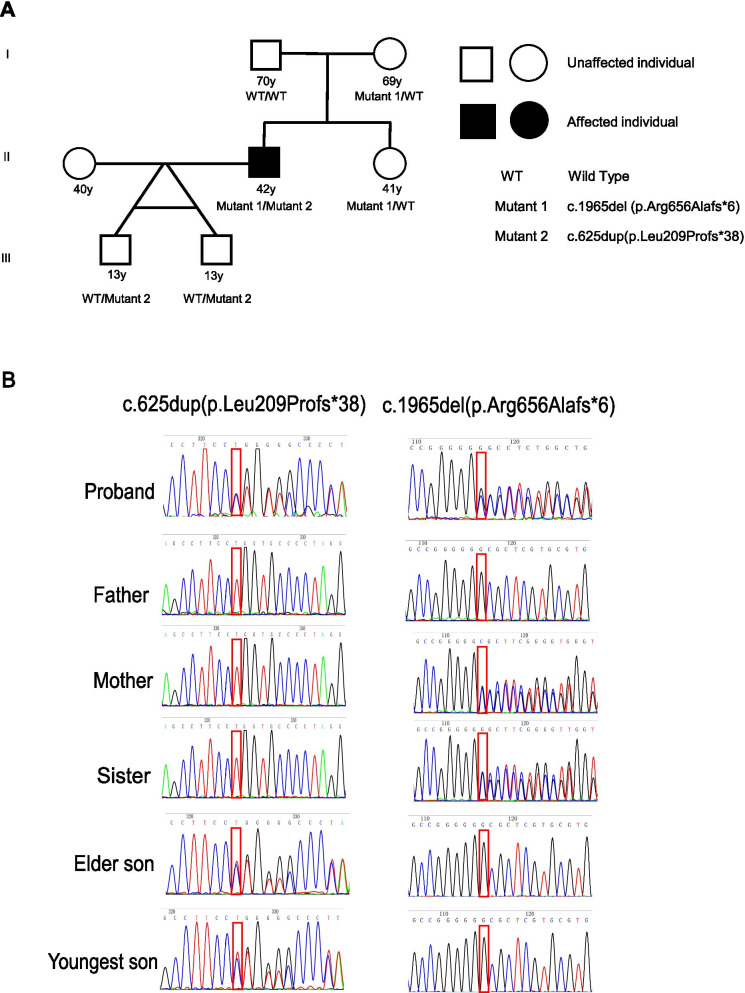
The pedigree and detection of mutations in AD2002. **A** The pedigree of AD2002 showed that his healthy father (I:1) did not carry mutations, and his mother (I:2) and sister (II:2) were both heterozygous for an *LTBP3* variant, c.1965del (p.Arg656Alafs*6), and did not show abnormal clinical characteristics. His healthy twin sons (III:1, III:2) both harbored the heterozygous variant c.625dup (p.Leu209Profs*38). **B** Chromatograms of Sanger sequencing of AD2002 and his family members. The mutations were all verified by Sanger sequencing

Patient AD2002 was a 42-year-old man, 165 cm/63 kg, with no hypertension history. He had sudden back pain during a business trip and went to the local hospital. Echocardiography showed dilatation of the aortic sinus (53 mm) and ascending aorta (38 mm), and thoracic computed tomography (CT) revealed aortic dissection in the descending aorta (DeBakey type III). TEVAR surgery was then performed. Two months later, he came to Fuwai Hospital for reexamination and a genetic test. After recognizing his two *LTBP3* mutations, we further asked about his symptoms and family history. He also had spinal stenosis and dental anomalies, for which his teeth were all replaced at the age of 38. Nevertheless, his mother and sister, as well as his two sons, who all harbored only one heterozygous variant, had no abnormalities (Fig. 1A). No other homozygous or compound heterozygous mutations in this gene were detected in our cohort.

Subsequently, rare heterozygous *LTBP3* mutations were filtered in our dataset, with a MAF< 0.00005 in gnomAD. A total of four missense mutations were detected in four individuals affected with early-onset thoracic aortic dissection (Table 2). None of these patients had significant systemic abnormalities other than the aortic disease (details in Table 2). None of the mutations were found in TAAD genes that had been approved by ClinGen in these patients, except for a variant of unknown significance (VUS) in the *FBN1* gene. Patient AD820 harbored the *FBN1* variant c.994C>T (p.Arg332Cys) and the *LTBP3* variant c.1588A>G (p.Thr530Ala), both of which remained uncertain significance.

**Table 2:** Phenotypic characteristics in sporadic patients with rare heterozygous LTBP3 variants in this study

Patient ID	Age (year)/gender	Height(cm)/body weight (kg)	Aortic disease	*LTBP3* mutations	Other clinical features	Variants in other HTAAD gene in ClinGen
AD721	52/M	172/75	Type B aortic dissection	c.1456G>A(p.Gly486Arg)	Hypertension; Chest pain; Carotid atherosclerotic plaque	No
AD820	41/M	171/80	Type A aortic dissection	c.1588 A>G(p.Thr530Ala)	Hypertension; Chest pain; Aortic regurgitation; Diabetes	*FBN1*, c.994 C>T(p.Arg332Cys)
AD977	48/M	170/80	Type B aortic dissection	c.1510G>A(p.Glu504Lys)	Hypertension; Chest pain; Aortic regurgitation	No
AD2076	33/M	173/95	Ascending aortic aneurysm and dissection	c.152 C>G(p.Ala51Gly)	Myopia	No

*LTBP3*, NM_001130144; *FBN1*, NM_000138

## Discussion

Disturbed TGF-β signaling has been extensively implicated in thoracic aortic aneurysm and dissection. Mutations in *FBN1* lead to defective ECM microfibrils and dysregulation of TGF-β signaling, both of which play a vital role in the development of aortopathy in MFS patients [23–27]. Several genes in the TGF-β signaling pathway, such as *TGFBR1, TGFBR2, SMAD2, TGFB2, TGFB2, and SMAD2*, have been proven to be causative for syndromic TAAD [28, 29]. The *LTBP3* gene encodes latent transforming growth factor-beta binding protein 3, which has a similar structure to *FBN1*. Both of them have multiple EGF-like repeats and unique 8-cys domains that are interspersed with TB domains [30]. *LTBP3* regulates TGF-β activity by enabling its secretion, directing it to specific sites in the ECM, and participating in its activation [31–34]. It is widely expressed, including in the skeleton, tooth, heart, and aorta, which suggests that mutations in this gene may lead to a broad range of phenotypes.

*Ltbp3*-deficient mice were initially documented to have small body sizes, unique craniofacial malformations, bone abnormalities, and dental anomalies [35–39], which were consistent with the clinical features in individuals with *LTBP3* mutations, recessive-pattern DASS, and dominant-pattern ACMCID and GPHYSD3 patients. In earlier reports, cardiovascular deformities were not described in either mouse model or patient. Subsequently, Zilberberg et al. [34] have shown that lack of *Ltbp3* could attenuate the aneurysmal phenotype and prevent premature death of *Fbn1*^mgR/mgR^ mice, probably by the reduced activation of TGF-β signaling. However, Guo et al. [10] pointed out that when aortic diameters were normalized by body mass, the diameter of the aortic root and ascending part in *Ltbp3*^−/−^ *Fbn1*^mgR/mgR^ mice was similar to that in the *Fbn1*^mgR/mgR^ group, and *Ltbp3* deficiency resulted in spontaneous aortic dilation. These data suggested that *Ltbp3* was associated with the formation and progression of thoracic aortic aneurysms, although the exact effect and mechanism were not well understood. It was reasonably assumed that *LTBP3* mutations might contribute to TAAD by disturbing the TGF-β signaling pathway and extracellular matrix assembly. More studies are needed to determine its specific role in TAAD.

Regarding the clinical data, there were only a few reports supporting the role of *LTBP3* in the cardiovascular system. Dugan et al. reported that two sisters in a single family with a homozygous truncated mutation of *LTBP3* both had mitral valve prolapse (MVP) [7]. Guo et al. reported that biallelic null mutations in *LTBP3* could predispose individuals to thoracic aortic aneurysms and dissections, and heterozygous rare *LTBP3* variants might be related to an early onset risk of acute aortic dissection [10]. This was the first and to date, the only report that supported *LTBP3* mutations that might be responsible for TAAD. Our study identified two compound heterozygous variants in *LTBP3* in an aortic dissection patient, along with short stature and dental problems. They were both frameshift mutations, which were predicted to result in nonsense-mediated mRNA decay (NMD). This was consistent with the inherited pattern and mutation types in DASS, and it offered more clinical evidence showing that *LTBP3* mutations could cause TAAD.

When reviewing the previously reported cases with homozygous or compound heterozygous *LTBP3* null mutations, it was not hard to find that the limited numbers of affected individuals were all diagnosed with DASS at an early age (Table 3). Cardiovascular manifestations were not observed and mentioned specifically. Therefore, we could not exclude the possibility that they might have aortic deformities for the rest of their life. It was reasonable and advisable to follow up these patients to see whether they would have aortic or valvular problems. As *LTBP3* was not included in the reevaluation list of the ClinGen expert group on familial TAAD genes [1], it was not incorporated into routine targeted genetic analysis and was mostly not taken into account as a key candidate even in WES. Thus, there was a need to reanalyze the existing WES data on TAAD patients, which might reveal more novel mutations in this gene. More data are needed to assess the frequency of aortic or valvular abnormalities in DASS, which might be more frequent than we thought.

**Table 3:** Clinical characteristics of DASS patients with LTBP3 mutations in our study and previous studies

Phenotype	Total	AD2002	Patient 1/2/3/4	Patient 5/6	Patient 7/8	Patient 9/10	Patient 11	Patient 12/13/14	Patient 15	Patient 16	Patient 17/18/19	Patient 20/21
Age (year)		42	30/28/39/41	18^1/6^//15^1/4^	14/5	13/5^1/2^	11	16/9/12	24	7	54/55/59	44/58
Gender		M	M/M/F/F	F/F	F/F	F/M	M	F/F/M	F	F	M/F/F	M/F
Ethnic		Chinese	Pakistan	Emirati	Turkey	Caucasian French	Brazil	Pakistan	Thai	Indian	American	American
Family		TAAD_2002	F1	F2	F3	F4	F5	F6	F7	F8	F9	F10
Dental anomalies												
Tooth missing	7/22	+	+/+/+/+	+/NA	NA/NA	NA/NA	NA	NA/NA/NA	−	+	NA/NA/NA	NA/NA
Amelogenesis imperfecta	15/22	NA	NA/NA/NA/NA	NA/NA	+/+	+/+	+	+/+/+	+	+	+/+/+	+/+
Skeletal system												
Short stature	22/22	+	+/+/+/+	+/+	+/+	+/+	+	+/+/+	+	+	+/+/+	+/+
Osteopenia	7/22	NA	−/−/−/−	NA/NA	+/+	NA/NA	NA	+/+/+	NA	−	−/−/+	−/+
Brachydactyly	5/22	−	−/−/−/−	±/±	NA/NA	+/+	NA	NA/NA/NA	NA	+	NA/NA/NA	NA/NA
Scoliosis	15/22	−	+/+/+/+	+/+	+/+	NA/NA	+	+/+/+	+	−	−/−/+	−/+
Cervical/Lumbar vertebral body abnormality	7/22	+	−/−/−/−	+/+	NA/NA	NA/NA	NA	+/+/+	NA	+	NA/NA/NA	NA/NA
Cardiovascular disease												
TAAD	5/22	+	NA/NA/NA/NA	NA/NA	NA/NA	NA/NA	NA	NA/NA/NA	−	−	+/+/−	+/+
MVP	5/22	−	NA/NA/NA/NA	+/+	N/ANA	NA/NA	NA	NA/NA/NA	?	−	−/+/+	−/+
*LTBP3* mutations		c.1965del (p.Arg656Alafs*6); c.625dup (p.Leu209Profs*38)	c.2322 C>G (p.Tyr774*)	c.1858_1859insG (p.Cys620Trpfs*171)	c.2071_2084del (p.Tyr691Leufs*95)	c.421 C>T(p.Gln141*); c.1531+1G>T	c.2216_2217del (p.Gly739Alafs*7)	c.2356_2357del (p.Val786Trpfs*82)	c.1721-2 A>G	c.3153_3154del (p.Cys1051*); c.689_690del (p.Val230Alafs*16)	c.132del (p.Pro45Argfs*25); c.2248G>T (p.Glu750*)	c.2033_2041delinsCTT (p.Asn678_Gly681 delinsThrCys)
Zygosity		Compound heterozygous	Homozygous	Homozygous	Homozygous	Compound heterozygous	Homozygous	Homozygous	Homozygous	Compound heterozygous	Compound heterozygous	Homozygous
References		This study	Noor et al. (2009) [40]	Dugan et al. (2015) [7]	Huckert et al. (2015) [39]	Huckert et al. (2015) [39]	Huckert et al. (2015)[39]	Huckert et al. (2015)[39]	Intarak et al. (2019)[8]	Kaur et al. (2020)[41]	Guo et al. (2018)[10]	Guo et al. (2018)[10]

*LTBP3*, NM_001130144; TAAD, thoracic aortic aneurysm and dissection; MVP, mitral valve prolapse; M, male; F, female; NA, not available; +, presence; −, absence; ±, equivocal; Patients 1–21 indicated that there were 21 patients reviewed; F1–10 suggested individuals from 10 different families

**Fig. 2:**
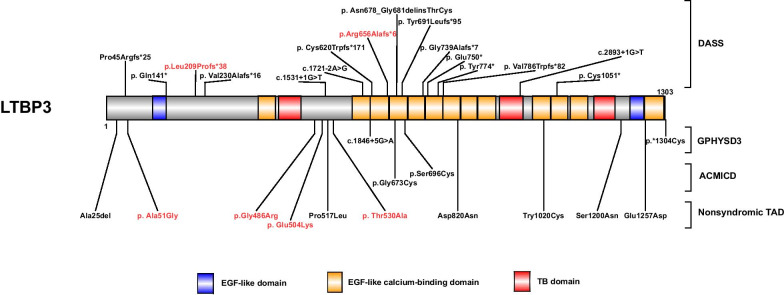
Schematic diagram of the *LTBP3* domain structure and mutation. The identified mutations were indicated above (DASS) and under (GPHYSD3, ACMICD, nonsyndromic TAD) the protein diagram. The mutations identified in this study were shown in red letters, while mutations reported by others were marked in black letters

A comprehensive summary of all reported *LTBP3* mutations is demonstrated in Fig. 2. It was observed that the majority of bi-allelic loss-of-function *LTBP3* mutations, which contributed to DASS, were present in the highly conserved EGF-like calcium-binding domain, while most of the rare monoallelic variants associated ACMICD, GPHYSD3, and thoracic aortic dissection (TAD) were missense mutations located in the same region, indicating that different inheritance modes and natures of *LTBP3* might lead to different diseases. Nevertheless, currently reported monoallelic variants in this gene are so limited that further investigations are needed to assess their pathogenicity and apply thorough evaluations to the patients.

The relationship between rare *LTBP3* heterozygous variants and aortic dissection risk also remains unclear. Most of the rare *LTBP3* variants detected in early-onset of aortic dissection patients by Guo et al. [10] were in the key EGF-like calcium-binding domains, while ours were not, which were less likely to be causative. However, it could not be ruled out that these variants had a mild effect on the development and/or severity of the disease, due to their high CADD predictive scores. Patient AD820 was identified with a variant with unknown significance (PM1, PP3) in the *FBN1* gene, c. 994C>T (p.Arg332Cys), which was reported at a low frequency (0.000008808) in the gnomAD database. At the same time, he was identified to carry an *LTBP3* heterozygous variant. There was a possibility that these rare variants contributed together to the disease. More cases are needed to fully describe the disease map and genotype-phenotypic association.

## Conclusions

In summary, we identified the first case of a TAAD patient with bi-allelic *LTBP3* frameshift mutations in an Asian population, as well as several rare *LTBP3* variants in affected individuals with early aortic dissection. This expanded the gene spectrum of *LTBP3* and provided more support for its role in TAAD. Our data show the necessity of incorporating this gene into the routine genetic analysis of aortic aneurysms and dissections, which would help to fully understand its phenotypic spectrum and raise the diagnosis rate of TAAD.

## Data Availability

All data generated or analysed during this study are included in this published article.

## Abbreviations

TAAD: Thoracic aortic aneurysm and dissection
WES: Whole exome sequencing
DASS: Dental anomalies and short stature
ACMG: American College of Medical Genetics
